# Gold Nanoparticle Radiosensitization for High-Dose-Rate Brachytherapy: Differential Efficacy of PEG- and RGD-Functionalized Gold Nanoparticles Across 2-Dimensional, 3-Dimensional, and in vivo Prostate Cancer Models

**DOI:** 10.2147/IJN.S599472

**Published:** 2026-06-12

**Authors:** Daniel Cecchi, Nolan Jackson, Dmitri Karaman, Wayne Beckham, Chris D Johnstone, Samantha A M Lloyd, Devika Chithrani

**Affiliations:** 1 Department of Physics and Astronomy, University of Victoria, Victoria, BC, Canada; 2 Department of Mechanical Engineering, University of Victoria, Victoria, BC, Canada; 3 Axolotl Biosciences, Victoria, BC, Canada; 4 Medical Physics Department, BC Cancer, Vancouver, BC, Canada; 5 Division of Radiation Oncology, University of British Columbia, Vancouver, BC, Canada; 6 Medical Physics Department, BC Cancer, Victoria, BC, Canada; 7 Centre for Advanced Materials and Related Technologies (CAMTEC), University of Victoria, Victoria, BC, Canada

**Keywords:** gold nanoparticles, high dose rate brachytherapy, radiotherapy, radiosensitization

## Abstract

**Purpose:**

Gold nanoparticles (GNPs) are promising radiosensitizing agents for radiation therapy; however, their implementation into high dose rate brachytherapy (HDR-BT) remains underexplored. Surface functionalization with polyethylene glycol (PEG) or integrin-binding domain RGD impacts their biodistribution and intracellular uptake, but whether active cellular targeting via RGD improves radiosensitization for HDR-BT has not been established in vivo.

**Methods:**

Here, we systematically compare the radiosensitization efficacy of non-targeted PEGylated GNPs versus actively targeted PEG-RGD GNPs on PC3 prostate cancer cells in vitro and in vivo, using clinically plausible concentrations, dosing procedures, and purpose-built irradiation platforms. 2-dimensional (2-D) and 3-dimensional (3-D) cell cultures, and male NRG mice were irradiated via a 192-Ir source delivered from a clinical HDR-BT afterloader. In vitro samples were dosed at 10 μg [Au]/mL and mice were intratumourally injected with 50 μL at 2 mg [Au]/kg bodyweight.

**Results:**

PEGylated GNPs did not elicit any radiosensitization either in 2-D, 3-D, or in vivo. RGD-functionalized GNPs elicited a 17% (*p=*0.001) reduction in survival fraction and 33% (*p*=0.005) greater DNA DSBs in 2-D cell cultures, and 57% (*p*<0.0001) reduction in 3-D spheroid growth compared to control samples 14 days post-irradiation. In a pre-clinical mouse xenograft model, tumour volume growth was also significantly reduced by 28% (*p=*0.005) 20 days post-irradiation compared to the irradiated control group, with no observable signs of acute toxicity from radiation delivery or administered GNPs.

**Conclusion:**

This research represents the first systematic in vitro and in vivo demonstration of GNP-induced radiosensitization using an HDR-BT source with clinically informed intratumoural delivery. Active cellular targeting with RGD functionalization was found to be a critical determinant of radiosensitization efficacy both in vitro and in vivo. Both GNP formulations demonstrated tolerability at the delivered dosing concentrations, and further research into the clinical deliverability of GNPs for HDR-BT is necessitated.

## Graphical Abstract

**Figure:**
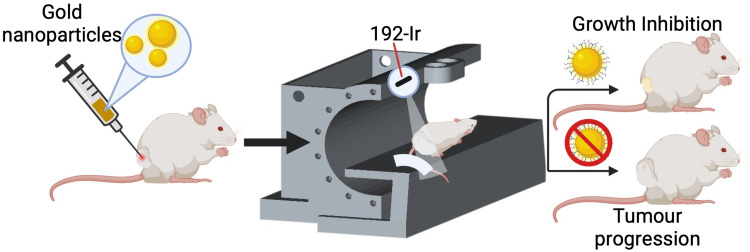

## Introduction

The current clinical application of brachytherapy for prostate, cervix, or breast cancer, continues to demonstrate its efficacy for achieving local tumour control as a boost to external beam radiotherapy (EBRT) or as monotherapy.^1^ Still to this end, treatment prescriptions must balance adequate target coverage with normal tissue exposure that can reduce its therapeutic efficacy.^2^,^3^ Improvements in treatment planning and delivery, such as MRI-guided needle placement and improved target delineation, can improve treatment delivery confidence but cannot fully address toxicity concerns.^4^ To improve local dose deposition and spare surrounding tissue, high-Z nanoparticles like gold, hafnium, and gadolinium are garnering increasing interest as effective interventions with conventional radiotherapy techniques.^5–7^ Gold nanoparticles (GNPs) are some of the most widely investigated platforms due to their facile synthesis, unique surface chemistry for facile functionalization, and biocompatibility and inertness.^8–11^ The high-Z nature of GNPs leads to a significantly greater photoelectric cross-section compared to normal tissue,^8–11^ leading to improved local dose deposition within treated tumours.^12–14^ Furthermore, they have been shown to be biocompatible with limited toxicity in vitro and in Phase I clinical trials.^15^ GNPs may be most advantageous for high dose rate BT (HDR-BT) with low to medium energy photon emission that takes advantage of a high photoelectric cross section and high dose rates, which would lead to a greater generation rate of cell-damaging species.

Despite the promise of GNPs in HDR-BT, their efficacy remains largely uninvestigated compared to other treatment techniques like EBRT due to the complexity of treatment delivery from an isotropic radioactive source delivered via guide channels to pre-defined dwell positions. The limited published work that does exist shows the potential of GNPs to increase DNA DSBs and reduce cellular survival.^16–19^ However, many of these studies are constrained by important limitations like simplistic biological systems, such as plasmid DNA rather than cell cultures, or dosing concentrations well above clinically plausible levels that limit their translational relevance.^16^,^18^ Our previous work addressed these limitations by developing a novel solid water phantom capable of uniform and reproducible irradiations to monolayer cell cultures, and clinically plausible GNP concentrations, and demonstrated significant GNP-induced radiosensitization in cervical and prostate cancer cell lines.^17^ Importantly, no published study has yet evaluated GNP radiosensitization in HDR-BT beyond simplistic 2-D cell culture systems, and the efficacy of intratumourally delivered GNPs in vivo remains entirely unexplored.

The effective delivery of GNPs into the target volume is another consideration for the clinical translation of GNPs into HDR-BT. While intravenous injections are likely to be favoured for non-invasive treatment techniques like EBRT, or poor anatomical accessibility of the tumour. However, intravenous injections lead to poor tumour accumulation of GNPs, even with surface functionalization that shields GNPs from phagocytosing elements like macrophages and circulating monocytes.^20–22^ Intratumoural (i.t.) injections could be a practical alternative for HDR-BT treatments, where invasive surgical procedures or introduction of applicators into body cavities are required for treatment delivery. Previous work shows that i.t. injections of GNPs naturally leads to greater tumour accumulation compared to intravenous injections, but this is also impacted by their functionalization status.^21^ Furthermore, i.t. injections have previously been clinically incorporated into previous radiotherapy workflows and is currently the standard practice for delivery of a novel metallic radiosensitizer, NBTXR3, with EBRT.^23^,^24^

**Figure 1:**
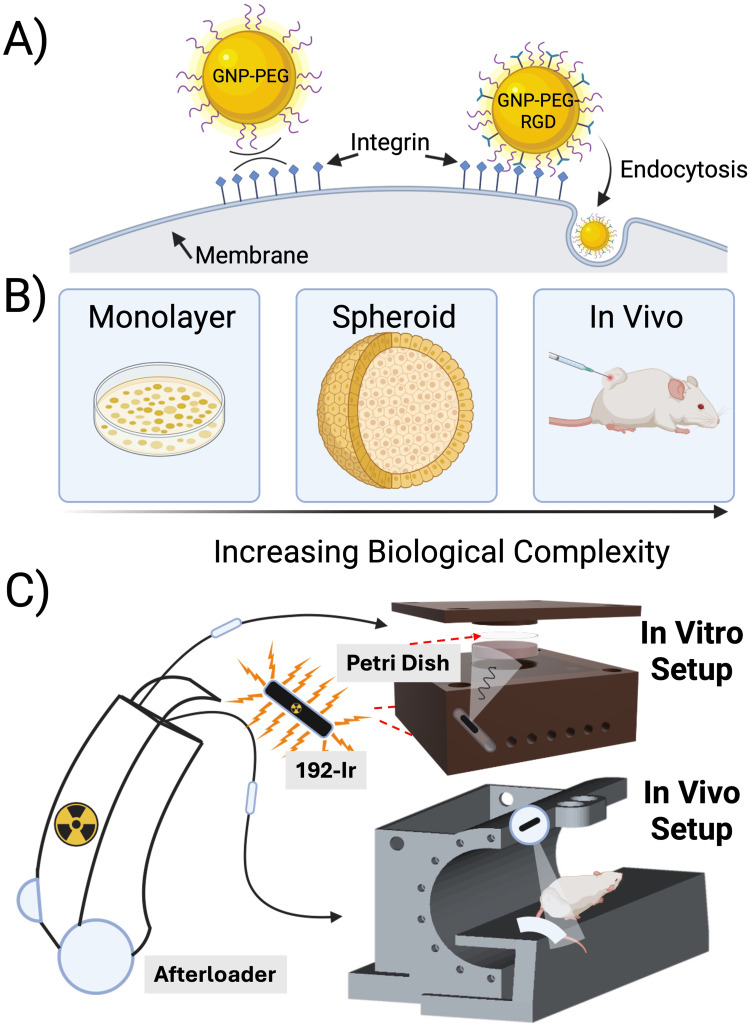
(**A**) Functionalization strategy of gold nanoparticles (GNPs) affects intracellular uptake kinetics. (**B**) Representative in vitro to in vivo systems increase in biological complexity, allowing more relevant assessment of GNP kinetics. (**C**) In vitro and in vivo radiation delivery accomplished using novel in-house irradiation jigs enabling uniform and repeatable treatments.

High tumour accumulation of GNPs following i.t. injection may not guarantee radiosensitization as their localization within the tumour cells or retention within the tumour stroma is largely unknown. Functionalization with polyethylene glycol (PEG) is associated with prolonged blood circulation due to its favourable shielding properties in vivo but PEGylation also shields the GNPs from endocytosis, reducing their intracellular uptake. Previous investigational studies that have introduced active cellular targeting through functionalization with integrin-binding domain RGD on top of PEGylation demonstrate greater tumour cell uptake and radiosensitization compared to non-targeted counterparts in vitro (Figure 1A).^11^,^25–28^ Intracellular localization of GNPs could be a critical determinant of their radiosensitizing properties due to their hypothesized impact on mitochondrial respiration and ROS generation.^29–31^ Whether the active targeting status of functionalized GNPs impacts their radiosensitive properties post-i.t. injections has not yet been investigated in vivo.

In this work, we compared the efficacy of non-targeted PEGylated GNPs versus actively targeted PEG-RGD GNPs in HDR-BT by systematically determining their radiosensitization properties on PC3 prostate cancer cells across 2-D monolayer cell cultures, 3-D tumour spheroids, and an in vivo xenograft mouse model (Figure 1B). In vitro and in vivo irradiations were accomplished using in-house built irradiation platforms developed specifically for uniform and reproducible HDR-BT dose delivery (Figure 1C). Male NRG mice were inoculated with PC3 tumours, intratumourally injected with the functionalized GNPs, irradiated, and then monitored for tumour growth and signs of acute toxicity. To the authors’ knowledge, this study is the first to directly compare the radiosensitization of targeted versus non-targeted GNPs in vivo using a clinical HDR-BT source and clinically informed i.t. delivery. While further investigations are necessitated to evaluate long-term toxicity of the GNP complexes, we demonstrate that i.t. injections of GNPs could be an efficacious and safe treatment delivery technique for future HDR-BT patients.

## Materials and Methods

### Synthesis and Analysis of GNPs

Spherical GNPs were synthesized following a citrate-reduction method.^8^ Briefly, 1.18 mL of 1% HAuCL_4_ ⋅ H_2_O (Sigma – Aldrich, St. Louis, MO, USA, catalog no. 520918) was added to 28.82 mL of double-distilled water in an Erlenmeyer flask. The solution was then brought to a boil while being stirred vigorously. 1.12 mL of 5% (w/v) sodium citrate (Sigma-Aldrich, catalog no. S4641) was then added rapidly to the boiling solution during continuous stirring. The solution was then left to mix for ~5–10 minutes until the colour turned a semi-opaque ruby red colour. The flask was then transferred to a room temperature stirring plate to equilibrate to room temperature while under continuous stirring.

Surface functionalization of the GNPs was accomplished first with a thiol-terminated 2kDa PEG (mPEG-SH; Nanocs, catalog no.: PG1-TH-2k) followed by a 1.6 kDa peptide containing integrin-binding domain RGD (CKKKKKKGGRGDMFG; Anaspec). PEG was added to the colloidal GNP solution with agitation at a concentration of 1 PEG molecule per nm^2^ of GNP surface area. The RGD peptide was subsequently added to achieve a final surface ratio of 2:1 PEG to RGD molecules.

The size and shape of the GNPs were verified using scanning transmission electron microscopy (STEM) (ultrahigh resolution STEM SU9000, Hitachi). The size and concentration estimates of the colloidal solution were measured using UV-vis spectrometry (PerkinElmer λ 365 Spectrophotometer). The stability and functionalization of the GNPs were determined by measuring the hydrodynamic diameter and ζ potential using a LiteSizer 500 particle analyzer.

### Monolayer Cell Culturing and Spheroid Growth

PC3 prostate cancer cells were purchased from the American Type Culture Collection (ATCC) (ATCC#: CRL-1435). The cell line was cultured in Dulbecco’s Modified Eagle Medium (DMEM; Gibco) supplemented with 10% Fetal Bovine Serum (Gibco), 1% Penicillin and Streptomycin (Gibco), and 4 mM GlutaMax (Gibco). Cells were incubated at 37 °C with 5% CO_2_ and subcultured once confluency reached approximately 70–80%. Cell washing and dissociation was achieved using phosphate-buffered saline (PBS) (Gibco, catalog no.: 10010023) and a 0.25% Trypsin-EDTA solution (Gibco, catalog no.: 2520072), respectively. Cells were then seeded into 35 mm petri dishes prior to experimentation at a concentration to achieve 70–80% confluency after 48 hours later.

Spheroid formation was achieved by supplementing cell suspension with 4% Matrigel (Corning, catalog no. 356234) while being kept on ice. Cells were then seeded into ultralow attachment 96-well plates (Corning, catalog no. 3474) at a predetermined concentration to form spheroids with a final size of 300–350 μm in size after 72 hours of growth. The microplates were then centrifuged for 10 minutes at 450 g at 4 °C. Spheroids were incubated at 37 °C with 5% CO_2_ for 72 hours prior to initiation of experiments.

### Animal Models and Tissue Preparation

Male NRG mice were purchased from the BC Cancer Research Institute Animal Resource Centre (ARC, Vancouver, BC, Canada). The right flank of mice was injected with 5×10^6^ PC3 cells subcutaneously in a volume of 50 μL using a 28-gauge needle. Once the average tumour volume reached approximately 200–250 mm^3^, mice received intratumoural (50 μL, 800 mg [Au]/L) injections of either GNP-PEG or GNP-PEG-RGD complexes, depending on the treatment group. In vivo biodistribution of the GNP complexes was evaluated at 24 hours post-injection. Organs, tumours, and blood were then harvested from the sacrificed mice. Blood was collected via cardiac puncture with a. 25-gauge needle and placed into a microtainer tube (K2 EDTA) for plasma. Blood samples from a single mouse from each treatment unit were processed for complete blood count (CBC). Liver and kidney function via ALT, AST, and BUN, as well as total electrolytes and proteins were reported. Mice randomly selected for irradiation were transported from the animal care facility to the HDR-BT treatment vault 24 hours after GNP complex injection and were delivered a 4 Gy prescription to the tumour volume. Treatment planning and setup is discussed further in In Vivo Irradiations.

### Quantification of Absolute Gold Accumulation and Retention

In vitro monolayer and spheroid cell cultures were treated with functionalized GNPs (either GNP-PEG or GNP-PEG-RGD) at a concentration of 10 μg/mL for 24 hours. Three independent samples per condition per timepoint were used for in vitro analysis. Cell cultures were then washed with PBS, dissociated, and counted using a hemocytometer. For in vivo sample preparation, samples were weighed, then blended in 2 mL of 0.25% Trypsin-EDTA using a hand-held homogenizer. Once blended, samples were diluted to 5 mL with double-distilled water. We analyzed five mice per group per timepoint.

For both in vitro and in vivo samples, approximately 100–500 μL was transferred to glass tubes and treated with 250 μL of aqua regia [3:1 molar ratio of HCl to HNO_3_ (VWR)]. Between 30k and 50k cells were analyzed for in vitro samples, and one-tenth of the homogenized tissue samples were used for in vivo analysis. Samples were then placed into a mineral oil bath held at a constant temperature of 90 °C and left for 1 hour (in vitro) or 2 hours (in vivo). In vitro samples were then treated with 100 μL of hydrogen peroxide then left in the oil bath for an additional 1 hour. All samples were then diluted to a 2.5% (v/v) acid content with double-distilled water and passed through a 0.2 μm filter (Sigma-Aldrich, catalog no. SLLG025SS). Inductively coupled plasma mass spectrometry (ICP-MS) (Agilent 8800 Triple Quadrupole, Agilent Technologies) was used to analyze the absolute gold content in each sample [ng Au/mL], which was then related to the GNPs present in each cell via the following formula:

$${{Gold\,Nanoparticles} \over {Cell}} = {{\left({Gold\,Concentration\,Per\,Sample\,\left[{{g \over {mL}}} \right]} \right) \cdot \left({Sample\,Volume\,\left[{mL} \right]} \right) \cdot ({N_A}\left[{{{atoms} \over {mol}}} \right])} \over {\left({Gold\,Atomic\,Mass\,\left[{{g \over {mol}}} \right]} \right) \cdot \left({Number\,of\,Cells} \right) \cdot {{Gold Atoms} \over {Gold\,Nanoparticle}}}}$$

In the above equation, the atomic mass of gold is 196.96657 g/mol, *N_A_* is Avogadro’s number: 6.022×10^23^ atoms/mol, and the number of gold atoms per nanoparticle is calculated as follows dependent on the size of the GNP:

$${{Gold\,Atoms} \over {Gold\,Nanoparticle}} = {{\left({Atoms\,per\,unit\,cell} \right) \cdot \left({Gold\,Nanoparticle\,Volume\,\left[{n{m^3}} \right]} \right)} \over {Unit\,Cell\,Volume\,\left[{n{m^3}} \right]}} = 4 \cdot {{{{4\pi {r^3}} \over 3}} \over {{a^3}}} = {2 \over 3}\pi {\left({{D \over a}} \right)^3}$$

Where D = 12 nm is the core diameter of the GNP and a = 0.408 nm is the unit length of a cell of gold atoms.

### Hyperspectral Image Acquisition

In vitro samples were cultured in the bottom of 6-well dishes containing glass coverslips. The cells were then treated with functionalized GNPs and incubated for 24 hours, after which, they were washed with PBS and fixed with 4% paraformaldehyde (PFA) for 20 min at 37 °C. Coverslips were then washed twice with PBS before being mounted into glass slides using ProLong Glass Antifade (Thermofisher, catalog no. P36985).

In vivo samples were prepared by fixing tissue samples in 10% neutral-buffered formalin and processed into paraffin using an automated tissue processor. The samples were then sliced into 4 μm sections on glass slides. Hyperspectral imaging (HSI) (CytoViva) of both in vitro and in vivo samples was accomplished using a darkfield microscope under a 60X objective lens.

### Live Cell Imaging

To visualize GNP distribution in spheroid cultures, GNPs were functionalized with a thiol-terminated 2 kDa Cy5 (ex/em: 633/650) labelled PEG. At the pre-determined time points, monolayer cell cultures and spheroids were dosed with the GNP complex. Monolayer cell cultures were seeded on a 35 mm glass coverslip-bottom dish (MatTek, catalog no. P35GCOL-1.5–14 C). Spheroids were transferred to a similar dish for imaging with minimal media to maintain stability but avoid aspiration. The samples were then imaged using a confocal laser scanning microscope (Zeiss LSM 980, Carl Zeiss Microscopy GmbH, Jena, Germany) under a 60X objective lens for 2-D cell cultures and 10X for spheroids.

### In vitro Irradiations

#### 2-D Monolayer

In vitro irradiations were accomplished using an Elekta Flexitron HDR Afterloader (Model: 136149, Elekta, VEENENDAAL, Netherlands) and in-house built modular Solid Water phantom to house 35 mm petri dishes. The solid water phantom has been validated for uniform dose delivery to the base of petri dishes in previous work.^17^ 2-D cell culture preparation was performed as described in Monolayer Cell Culturing and Spheroid Growth. Cell cultures were seeded for either analysis via *clonogenic assay* or a *DNA double-strand break (DSB) assay*. Twenty-four hours before irradiation, cell cultures were dosed with functionalized GNPs at 10 μg/mL. After irradiation, media was removed from the monolayer cell cultures, which were then washed twice with 1 mL of PBS, and 3 mL of fresh media was added back to each well. The samples were immediately prepped for analysis and post-processing or left in the incubator for a predetermined time. Irradiated or unirradiated samples that are not treated with GNPs are labelled as control (CTRL).

#### Clonogenic Assay

Irradiated and unirradiated (sham) samples were carefully washed three times with PBS and trypsinized for cell counting. The required number of cells was then seeded to a 60 mm Petri dish in triplicate from a single-cell culture. Cells were plated at 100, 300, 500, 1000, 3000, 7000, 50000, 100000 per plate for 0, 1, 2, 3, 4, 5, 6, and 8 Gy, respectively. Post-irradiation, the seeded cell cultures were left to incubate for 15 days for colony growth. After the incubation period, the media was poured out, and the cells were stained with 0.5% methylene blue for 20 minutes, after which the stain was washed with dH_2_O, and the plates were allowed to air-dry overnight. Using a 10X microscope, colonies were counted for plating efficiency (PE) and survival fraction (SF) statistics. GNP-induced radiosensitization was evaluated using the sensitization enhancement ratio (SER) defined below:

(1) $$SER = {{Dose\,for\,25{\rm\%}\,SF\,w/o{\ }GNPs} \over {Dose\,for\,25{\rm\%}\,SF\,w/ GNPs}}$$

whereby the dose for each condition was determined using a fitted linear-quadratic (LQ) model.

#### Radiation-Induced DNA DSB Assay

For DNA damage analysis, cells were seeded onto glass coverslips within the 35 mm petri dishes and dosed according to Monolayer Cell Culturing and Spheroid Growth. DNA double-strand break (DSB) induction was evaluated 24 hours post-irradiation to 2 Gy. The specific assay and antibody staining protocol follows previously published research and will not be repeated here, though can be found in more detail in the Supplemental Material.^17^,^32^ Briefly, the 53BP1 repair protein localized to sites of DNA DSBs was stained with Alexa Fluoro 488 nm and the cells were imaged on glass microscope slides mounted with ProLong™ (P36930; ThermoFisherScientific, Waltham, MA, USA) Glass Antifade Mountant using a 60X oil immersion lens and a confocal laser scanning microscope (Zeiss LSM 980). Images were processed, and foci and nuclei were counted. GNP-induced DNA DSB-enhancement was quantified via the relative increase in average DSB foci-per-cell. Approximately 250–300 individual cells over 3–4 images were counted for each condition.

#### 3-D Spheroid

To successfully house and irradiate individual spheroids in 35 mm petri dishes, a PDMS insert for the petri dishes was cast using a 3-D printed mold. The Z_eff_ of PDMS is approximately 10.4, with a material density of 0.96 g/cm^3^.^33^ The insert contained eight symmetrically placed wells (5-mm diameter, 3-mm depth, 1-mm base) to hold individual spheroids in media. Dose delivery was evaluated using Monte Carlo (MC) simulation package TOol for PArticle Simulation (TOPAS) v3.9, with the phantom modeled as a 10×10×4 cm water block and the insert defined as a 3.5×3.5×1.0 cm PDMS volume.^34^ The box was voxelized (50x50x100 bins) to determine dose fall-off through the spheroids (~350 μm diameter) and at the height of the insert base. Each dwell position and time was simulated according to the treatment plan generated by the treatment planning system Oncentra^®^. The number of particle histories at each dwell position was changed according to the dwell time and the apparent activity (A_app_) of the source at the time of treatment plan delivery using the following formula:

$$Num.\,Particle\,Histories = {T_{Dwell }}\,\left[s \right] \cdot {A_{app}}\,\left[{Bq} \right]$$

The radial-dose fall-off was determined along the central axis of the insert and averaged within a radius of 2.5 mm.

Radiochromic film dosimetry was also performed with EBT-4 film (Lot #: 11212401) cut to fit within the 35 mm petri dish and placed below the molded insert before irradiation to 5 Gy. 24 hours post-irradiation the film was read in the red channel using an Epson Expression 10000XL (Epson, Suwa, Japan) and processed on using Python 3.11.2 on a personal computer. The film was calibrated using a calibration curve generated from a clinical 6 MV photon beam using the same film lot and calibration doses ranging from 0 to 10 Gy. Dose uniformity across the film was determined by measuring the mean dose (with its standard deviation) in a square region centred on the medial location of the film and not overlapping with the film edges or orientation mark.

3-D cell cultures were formed as described in Monolayer Cell Culturing and Spheroid Growth. Spheroids were dosed at 10 μg/mL 24 hours prior to irradiation. Eight individual spheroids per condition along with 100 μL of media were carefully transferred to each of the eight wells in an autoclaved insert that was placed into a 35 mm petri dish. The spheroids were then irradiated to a dose of 4 Gy. Post-irradiation, the spheroids were transferred back to ultralow attachment 96-well plates and their growth was monitored over the following 14 days via brightfield imaging using a BioTek Cytation 1 Multimode Reader (Agilent Technologies, Santa Clara, CA, USA).

### In vivo Irradiations

48 male NRG mice were purchased from the BC Cancer Research Institute Animal Resource Centre (ARC, Vancouver, BC, Canada). The right flank of the mice was injected with 5×10^6^ PC3 cells subcutaneously in a volume of 50 μL using a 28-gauge needle. The mice were inoculated at a time such that once the tumours reached approximately 250–300 mm^3^, the 192-Ir source activity was sufficiently high to allow for efficient treatment delivery (see Table S1 for range of treatment delivery times at varying source activities). The mice were then randomly assigned into one of six treatment groups (N = 8 per group) according to whether they received i.t. injections of either GNP-PEG, GNP-PEG-RGD, or no injection, and whether they received a dose of 4 Gy to the tumour.

In vivo treatment delivery was accomplished using an in-house built radiation jig capable of accurate and repeatable dose delivery to a defined target volume using a radioactive source delivered via an HDR-BT afterloader. Its main designis as follows: a 3-D-printed semi-circle with eight equally spaced holes for catheters connected to a HDR-BT afterloader surrounds the lateral side of a translatable treatment bed; an axial and sagittal laser mounted at the mid-point of the top of the semi-circle coincide at the jig’s centre to aid in tumour localization; a separate animal support device (couch) that can be adjusted independently for ease of animal setup.

The 3-D printed jig was CT-scanned with a PLA moulded mouse model with representative tumour on its left flank positioned on its couch, and without the laser hardware mounted. A helical scan was acquired on a GE Discovery RT scanner (GE Healthcare Technologies Inc, Chicago, IL) using 0.06 cm slice thickness, 40 cm field-of-view, 440 mA and 80 kVp. The scan was registered to a CT scan of the tumour-bearing mouse to contour representative organ volumes, and the model mouse scan with volumes was imported into Oncentra^®^ for planning. A 5 mm isotropic margin was added to the gross tumour volume to account for setup variation and tumour size variability between animals. A prescription normalization point was digitized at the medial most extent of the tumour along the axial slice in line with the centre of the jig and used for planning. We have previously shown sufficiently accurate and sufficiently uniform dose delivery using the jig in a technical report that is accepted to Medical Physics (Cecchi et al, Medical Physics, 2025).

The mice in the irradiation group were transported to the cancer centre in covered cages after patient-treatment hours to limit viewing of the animals. The entrance to the HDR-BT treatment unit was covered with a barricade to prevent patients or other staff from observing the animals. The jig was placed on top of a high friction mat to prevent slipping, which was all placed within a plastic container as a precaution to prevent the mice from escaping. Eight holes were drilled into the box to allow for the catheters to be connected to the afterloader via transfer guide tubes.

Mice were anaesthetized one at a time approximately 3–5 minutes before planned irradiation and were then individually brought into the treatment vault. Animals were anesthetized with a cocktail of alfalaxone/xylazine (37.5mg/4.5mg/kg) via intraperitoneal injection and monitored using a Brachytherapy Procedure Record during the procedure and until recovery. After radiation, animals were reversed with an equal volume of 0.1 mg/kg atipamezole. Each mouse was individually placed onto the treatment bed, and its tail was taped down to prevent movement should it prematurely recover from the anaesthetic. Once positioned, the bed was translated to localize the tumour to the centre of the jig using the onboard lasers. Post-irradiation, the mice were brought back to the cages where the animal care specialist carefully monitored their behavior until the anaesthetic wore off. After all irradiations were complete, the mice were transported back to the animal care facility for monitoring of tumour volume, bodyweight, and signs of acute toxicity. Mice were euthanized once the tumours reached approximately 800 mm^3^ or the mice were designated for moribund euthanasia due to other reasons at the discretion of the animal care specialists. Mice were terminated by isoflurane anaesthesia followed by CO_2_ asphyxiation. For both tumour volume and bodyweight curves, each animal’s data contributed to the group mean up to the time of euthanasia or study endpoint. Animals’ data were censored at their last measurement and did not contribute to group averages beyond that timepoint.

### Statistics

In vitro experiments were conducted in triplicate to attain statistical significance estimates. Error bars on individual sample measurements are defined as ±1σ. Clonogenic survival data were fitted to the LQ model describing cell survival fraction as a function of dose (D):

$$ SF = {e^{ - D\left({\alpha - \beta D} \right)}}$$

The α and β parameters were estimated for each group after the data was fitted using a non-linear least-squares method that employed a Levenberg–Marquardt algorithm (Scipy.optimize, v1.16.1). A parametric bootstrapping procedure was performed to calculate the 95% confidence interval (CI) of the parameters, along with the SER (defined in Equation 1). One thousand iterations created a synthetic dataset, from which a distribution of α, β, α/β, and SER values was created. The LQ parameters and SER will be reported from the measured dataset, and not the mean of the synthetic, bootstrapped dataset; however, the 95% CI from the bootstrapped distribution will be given.

The significance of the SER value will be concluded if SER = 1 is not contained in the 95% CI generated from the bootstrapped distribution. Similarly, to compare radiosensitization between individual groups, the difference in SER (Δ) was computed for each bootstrap iteration between paired groups. A 95% CI was obtained as described previously, and statistical significance was concluded if Δ = 0 was not contained in the interval. A two-sided p-value was then calculated as twice the fraction of differences that are greater or less than SER = 1 or Δ = 0. Statistical significance was defined as α = 0.05.

The statistical significance of GNPs on DNA DSB induction was achieved using a two-tailed Student’s *T*-test with equal variance on the measured foci/cell averages per condition. Statistical significance was defined as α = 0.05.

Tumour growth trends and mice bodyweight differences were compared between groups using an ordinary least squares multiple linear regression model with an interaction term. The group, day, and interaction term (group × day) were fixed effects. The model compared both the effects of group and day, as well as whether the rate of tumour growth or body weight change differed between groups. Endpoint-free survival of the two mice groups was evaluated using a Log rank test, where the defined endpoint event was planned euthanasia upon tumour volume reaching 800 mm^3^. Mice that had not reached this tumour volume by the end of the study period were censored at their last measurement. Significance was defined as α = 0.05.

## Results and Discussion

### Synthesis and Functionalization of GNPs

**Figure 2:**
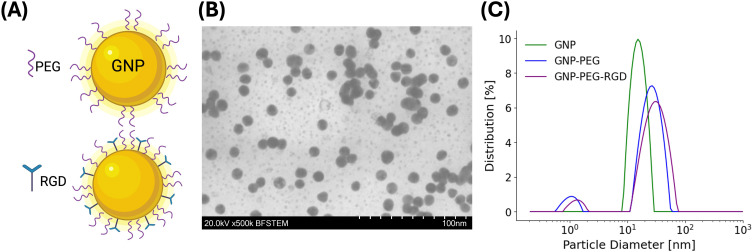
(**A**) Surface functionalization strategy first incorporating PEG as a stabilizing agent followed by RGD for tumour-specific targeting. (**B**) Transmission electron microscopy image of citrate-capped GNPs. (**C**) DLS stability measurements of bare and functionalized GNPs.

Spherical GNPs with a core diameter of 12 nm were synthesized, functionalized with PEG and RGD (Figure 2A) and confirmed via Transmission Electron Microscopy (TEM) (Figure 2B). 2 kDa PEG followed by a 1.6 kDa peptide containing integrin-binding domain RGD was surface functionalized to the GNPs to improve blood circulation in vivo and improve intracellular uptake.^11^,^35^,^36^ PEG was grafted at 1 PEG/nm^2^ of GNP surface area at a 2:1 ratio of PEG to RGD.^37^,^38^ Dynamic light scattering (DLS) verified the stability of the nanoparticle complex (Figure 2C). Ultraviolet visible spectrometry (UV-VIS) and Zeta potential measurements were additionally conducted at each step of the functionalization process (Figure S1).

Raw measurements of DLS, ζ, and UV-VIS maximum absorbance is shown in Table 1. The functionalization of the GNPs with both PEG and RGD increased the nanoparticle’s hydrodynamic diameter from 16.4 nm ± 0.2 nm to 31.0 nm ± 0.7 nm, with a polydispersity index of 9.6% ± 0.8% and 21.1% ± 0.5%, respectively, in accordance with previously published research.^32^,^39^ Small (~1 nm), unaggregated gold clusters are likely observed in GNP and GNP-PEG-RGD solutions based on the measured DLS spread (Figure 2C), though are not expected to significantly affect our results due to their low abundance compared to larger spherical GNPs. Upon functionalization with neutral PEG and RGD, the zeta potential is shifted from −36.2 ± 0.8 mV for bare GNPs to −4.7 ± 0.2 mV with functionalized GNPs. The measured absorbance of the nanoparticle solutions is measured using UV-VIS and can be related to the size and concentration of the GNPs.^40^ For citrate-capped GNPs, the spectral absorbance peaked at 519.8 ± 3.1 nm, which was red-shifted to 524.8 ± 4.3 nm with the functionalization with PEG and RGD. The stability of the GNP complexes in cell culture media has previously been demonstrated by Jackson et al^32^ and Bromma et al^41^ Further validation for their synthesis could be achieved using XRD analysis to observe the diffraction pattern of the GNPs; however, this technique was not accessible for this study, though may be investigated in future experiments.

**Table 1:** GNP Characterization During the Functionalization Process Measuring the DLS Peak, Peak of Zeta Potential, and Wavelength of Maximum Absorbance from UV-VIS

GNP	DLS [nm]	Zeta [mV]	UV-VIS [nm]
Bare	16.4±0.2	−36.2±0.8	519.8±3.1
PEG	27.4±0.2	−4.5±0.2	521.9±1.4
PEG-RGD	31.0±0.7	−4.7±0.2	524.8±4.3

### In vitro Nanoparticle Uptake

**Figure 3:**
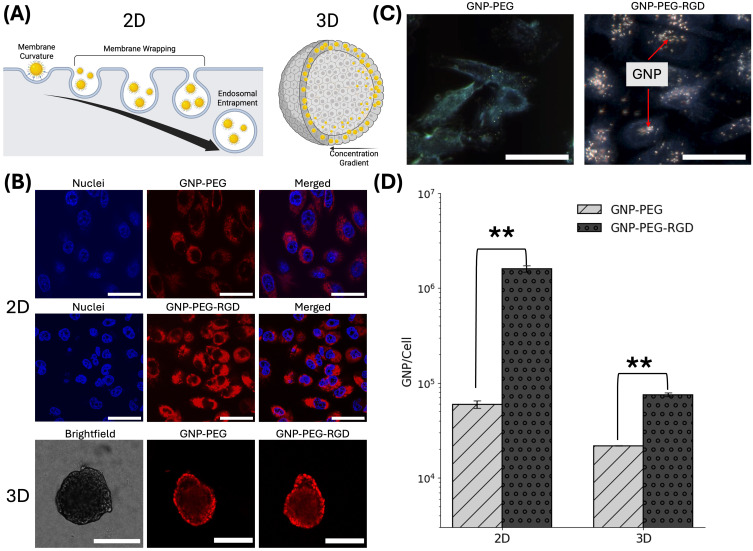
(**A**) Nanoparticle uptake and distribution in 2-D and 3-D cell culture models. (**B**) Live-cell imaging of 2-D monolayers (top) and 3-D spheroids (bottom) of both functionalization strategies following 24 hour dosing. (**C**) Darkfield images depicting intracellular distribution of GNPs. (**D**) Absolute GNP content per cell under both functionalization strategies for 2-D and 3-D cell cultures. *Scale bar 2-D: 20 μm; 3-D: 300 μm*; **: 0.001 < *p* < 0.01.

Intracellular uptake of GNPs primarily occurs via Clathrin-mediated endocytosis facilitated via RGD-functionalization (Figure 3A).^42^,^43^ Live-cell imaging depicting intracellular distribution of functionalized GNPs is shown in Figure 3B. Hyperspectral darkfield images were also taken of GNP-PEG and GNP-PEG-RGD-treated monolayer samples and can be found in Figure 3C. Absolute GNP content per cell is shown in Figure 3D. RGD-functionalization resulted in a 27-fold (*p* = 0.003) and 3.5-fold (*p* = 0.002) increase in intracellular GNPs 24 hours post-incubation in 2-D and 3-D tissue models. Notably, 2-D monolayer samples exhibited 21-fold (*p =* 0.003) and 2.7-fold (*p* = 0.004) greater intracellular uptake compared to their 3-D counterparts for GNP-PEG and GNP-PEG-RGD, respectively. This is likely a result of poor spheroid penetration by the nanoparticle complexes through the stiff extracellular matrix of the spheroid. We verified this result using live-cell imaging of tumour spheroids (Figure 3B), which indeed shows a concentration gradient within the spheroid as expected, similar to previous results.^20^,^21^

Many functionalizing strategies have been well-documented to boost GNP accumulation in vivo.^44^,^45^ The addition of PEG as a shielding strategy from serum protein absorption during blood circulation is one of the most widely documented techniques.^36^ However, this strategy results in significantly decreased cellular uptake, given that PEG shields the nanoparticle from engaging in receptor-mediated endocytosis, the predominant mechanism behind GNP uptake.^42^ As a countermeasure, we chose to functionalize the GNPs with RGD, which recognizes numerous integrin dimers expressed on the surface of cancer cells.^46^ Furthermore, the 12 nm diameter GNPs exhibit a high surface curvature compared to larger NPs, which improves receptor-ligand interactions and boosts RGD’s targeting efficiency when dual-functionalized with PEG.^47^

### In vivo Biodistribution

The absolute GNP content of both nanoparticle complexes showed significant tumour uptake at the 24 hour time point.^21^ RGD-functionalized nanoparticles exhibited slightly greater uptake compared to the PEGylated counterparts, likely due to lower stromal diffusion from interactions between the RGD ligand and extracellular matrix (ECM) components, restricting their release into the blood stream and sequestration in other organs; however, this was not statistically significant (*p* = 0.39). RGD functionalized nanoparticles have a higher chance of re-entry into the same or other neighbouring cells, which could lead to greater measured retention of the GNPs over time.^48^ A significantly larger (*p* = 0.006) gold content in the blood plasma is measured with GNP-PEG compared to GNP-PEG-RGD, which is likely a result of increased blood circulation time of PEGylated GNPs, while GNP-PEG-RGD get phagocytosed rapidly due to the recognition of the RGD sequence by circulating monocytes.^20^,^49^,^50^ We also investigated the retention of GNPs as a function of time shortly after injection and found evidence for high secretion of the GNP-PEG complex into the blood stream within 8 hours compared to GNP-PEG-RGD.^21^ We hypothesized that due to the positively charged RGD, lower stromal diffusion through and out of the tumour occurs, improving tumour retention and probability of cellular internalization by exocytosis.

**Figure 4:**
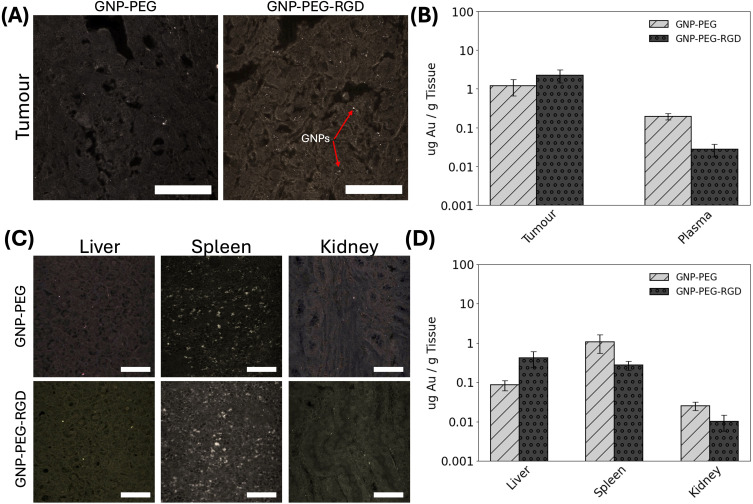
(**A**) Histology images of tumour sections showing GNP uptake. (**B**) Absolute gold content per gram of tissue in tumour and in blood plasma. (**C**) Histology images of liver, spleen, and kidney. (**D**) Absolute gold content per gram of tissue in the organs. *Scale bar is 40 μm*.

GNP distribution within the extracellular media was observed using darkfield images of histological samples of treated tumours (Figure 4A). The images confirm high localization of the GNPs within the tumour stroma, though more GNPs were observable in the GNP-PEG-RGD group. Absolute gold content in the tumour and blood plasma is shown in Figure 4B and confirms high localization of both functionalization strategies in the tumour. However, greater GNP-PEG accumulation was found in the blood plasma compared to GNP-PEG-RGD, likely due to greater secretion from the tumour stroma with only PEGylation that we have previously confirmed in other work.^21^ It is unclear from both ICP-MS and darkfield images whether GNP-PEG treated tumours resulted in the same levels of intracellular GNPs as those treated with GNP-PEG-RGD, or whether the GNPs remained within the extracellular medium without cellular internalization.

Absolute GNP uptake in organs post-i.t. injection is shown in Figure 4C and D. Tissue histology images of the nanoparticle complexes in Figure 4C confirm high localization in the spleen, which is confirmed with the measured gold content in Figure 4D. More GNP-PEG-RGD is measured in the liver compared to GNP-PEG, which is likely a result of up-regulated phagocytosis due to the RGD functionalization. Poor kidney accumulation with both functionalization strategies is reported as expected due to the size of the GNPs (~12 nm) being larger than can penetrate kidney tissue. Additional tissue histology images are provided in Figure S2.

The difference in qualitative GNP content between the PEGylated and RGD-functionalized GNPs in Figure 4A should be interpreted in the context of the absolute ICP-MS measurements in Figure 4B, which represent the quantitative measure of tumour gold content. The tissue histology images in Figure 4A may not accurately reflect the absolute GNP content in the tumour based on where the histology slides were chosen within the total tumour volume, as GNP diffusion through the tumour stroma is likely highly heterogenous. The greater liver accumulation of RGD compared to PEGylated GNPs in Figure 4C and D is likely attributable to recognition of the RGD sequence by Kupffer cells and liver sinusoidal endothelial cells, promoting receptor-mediated phagocytosis and sequestration. This is consistent with published literature demonstrating that RGD-functionalized nanoparticles exhibit enhanced uptake by MPS components including Kupffer cells in the liver.^20^,^49^,^50^ ICP-MS quantification in Figure 4D could support that the GNPs are preferentially accumulating within these Kupffer and endothelial cells, which are processed within the ICP-MS analysis rather than within liver tissue that can be visualized via darkfield microscopy in Figure 4A.

Liver and kidney toxicity biomarkers are presented in Table S2 for a single mouse from each treatment unit. In the GNP-PEG treated mouse, no elevated levels of AST, ALT, BUN, and Albumin were observed. However, the GNP-PEG-RGD treated mouse showed elevated ALT and AST markers possibly indicating acute liver inflammation, though did not show a meaningful difference in other markers relative to the GNP-PEG mouse or from reference conditions.

### 3-D Spheroid-Insert Dose Validation

**Figure 5:**
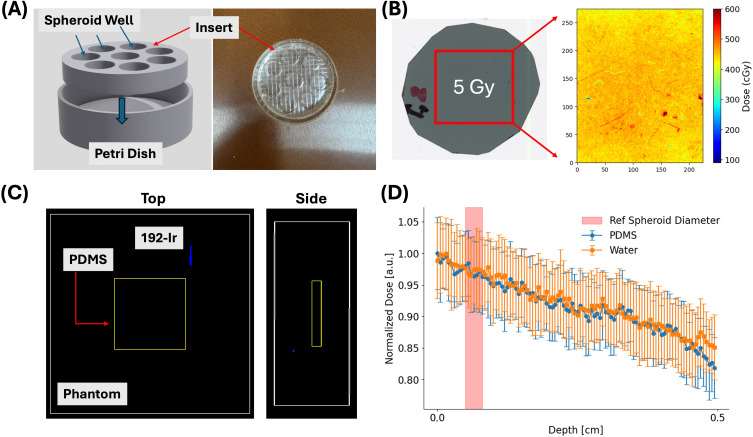
(**A**) Model of spheroid insert and photograph within petri dish and solid water phantom. (**B**) Irradiated film to 5 Gy (left) along with read film in the red channel (right). (**C**) Monte Carlo simulation setup depicting 192-Ir, PDMS insert, and Solid Water phantom. (**D**) Dose fall-off through the centre of the insert from central axis with PDMS and Water insert material.

A novel solid water phantom capable of uniform irradiations to the base of a 35 mm petri dish was previously described by Cecchi et al,^17^ which is effective for 2-D monolayer cell cultures. However, to enable 3-D spheroid irradiations a novel insert was developed to fit within the 35 mm petri dish, a PDMS insert was designed to house eight symmetrically placed spheroids near the base of the petri dish (Figure 5A). To ensure the insert and height of the spheroids did not significantly affect the delivered dose to the spheroid, both radiochromic film irradiations and MC simulations were conducted.

EBT-4 radiochromic film placed beneath the insert measured an average dose of 4.46 Gy ± 0.19 Gy, approximately 10% less than the 5 Gy prescription (Figure 5B). We hypothesize that the ~50 cGy dose difference in the measured film may be due to small air gaps introduced by placing the insert on top of the film that could correspondingly reduce the amount of scatter and thus the dose to film. Absolute dosimetry is accomplished using a 6 MV calibration curve as the energy dependence on EBT-3 dose measurement using a 192-Ir source was previously found to be less than 4% above 2.5 Gy.^51^ The primary purpose of this dosimetric validation was to confirm that the PDMS insert did not significantly affect the dose delivered to the spheroids. Since all comparative analyses are performed between spheroid treatment groups irradiated under identical conditions, the ~10% deviation from the nominal prescription does not affect the interpretation of the radiosensitization results. To determine whether the height of the spheroids due to the base of the insert affected the dose to the spheroids, we simulated the depth-dose fall-off from the 192-Ir source through the petri dish and media (Figure 5C). Dose fall-off was linear with depth at approximately 0.04%/0.1 mm in PDMS and 0.03%/0.1 mm in water (Figure 5D). The average diameter of the spheroids at the time of irradiation is approximately 350 μm, or 0.35 mm. The average height of the base of the insert was measured as 1 mm, which would reduce the delivered dose at the base of the spheroid by 0.4% within the PDMS insert, according to the MC results. The diameter of the spheroids at this height would then lead to an approximate decrease in dose of 0.6%, from its base to its top. This small decrease in dose is not expected to significantly impact the results of the spheroid irradiations.

### In vitro Radiosensitization

**Figure 6:**
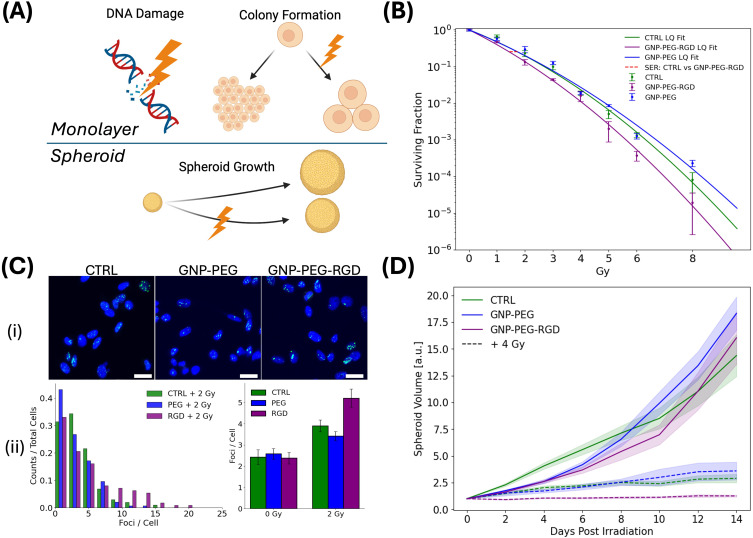
(**A**) Cell viability was quantified in 2-D using a proxy for DNA DSB damage and cell colony formation, and in 3-D measuring spheroid growth over time. (**B**) Clonogenic survival of GNP treated and untreated cells irradiated between 0–8 Gy. (**C**) Confocal images of DNA DSB damage in green and nuclei in blue of irradiated cells (i); DNA DSB foci / cell counts (ii). (**D**) Growth of irradiated and unirradiated spheroids up to 14 days post-irradiation; shaded region represents ±1 SEM. *Scale bar is 20 μm*.

We measured GNP-induced radiosensitization in vitro in both 2-D and 3-D cell cultures using common cell viability techniques (Figure 6A). At the time of irradiations, the apparent source activity was 7.6 Ci, corresponding to an averaged dose rate of approximately 0.9 Gy/min. Neither nanoparticle complex was observed to induce significant toxicity in cell cultures, indicating the viability of the dosing concentration. The plating efficiency (sham irradiation) of cell cultures dosed with or without GNPs was not found to be significantly different, along with no additional DNA damage reported via the incubation of these complexes (Figure S3).

Clonogenic survival of monolayer cell cultures is shown in Figure 6B with DNA damage response in Figure 6C. The functionalization strategy of the GNPs affected their measured radiosensitization. PEGylated GNPs with no additional RGD-functionalization did not exhibit any significant SER on monolayer cell cultures (SER = 1.00, 95% CI: 0.95, 1.25; *p* = 0.26). Similarly, no increase in DSB foci/cell post-irradiation was measured using the GNP-PEG complex (Avg. CTRL: 3.9 ± 0.3 foci/cell; GNP-PEG: 3.4 ± 0.3 foci/cell; *p* = 0.9). On the other hand, RGD-functionalized GNPs exhibited a significant SER of 1.2 (95% CI: 1.08–1.36; *p* = 0.001), coupled with a significant increase in DSB foci/cell (Avg. GNP-PEG-RGD: 5.2 ± 0.4 foci/cell; *p* = 0.005). These results follow the measured intracellular uptake of the functionalized GNPs, whereby lower uptake of the nanoparticles without RGD-functionalization resulted in poor endocytosis, which appears to have led to insignificant radiosensitization.

Regarding the increased DNA DSB frequency, based on the histogram shown in Figure 6C(ii), the increased DNA damage reported by the GNP-PEG-RGD complex likely results from a large increase in a subset of the cellular population, evident above ~10 foci/cell. It is currently unknown whether these cells simply exhibited higher uptake compared to others or if additional radiosensitization mechanisms are occurring. Endocytosis of nanoparticles is known to be highly heterogenous, which is a possible explanation for this effect; however, this will be investigated in future research.

Damage fixation occurred 24 hours post-irradiation to measure residual DSBs, which often go unrepaired, leading to loss in clonogenic potential.^52^ However, this timeframe leads to confusion regarding whether the GNPs are leading ot greater DNA damage or inhibiting DNA repair kinetics. The choice of GNP size of approximately 12 nm was chosen to prohibit their penetration into the nuclear membrane, limiting their direct impact on DNA repair kinetics. However, this effect cannot be completely ruled out with the current assay as GNPs can have an indirect effect where DNA repair is inhibited through the overproduction of ROS that could quench glutathione concentrations.^53^ The current assay technique would be challenging to use during earlier fixation time points because of damage saturation that would be difficult to quantify during imaging protocols; therefore, future investigations determining more complex kinetics between DNA damage, repair, and GNPs, could benefit from cytometry techniques measuring total fluorescence per cell, which would make it easier to quantify early DNA damage onset and facilitate a time-course analysis on the complex interplay between these three factors.^54^

The impact of GNPs on cellular survival can also be quantified using the α and β LQ model parameters. The α parameter is often interpreted as damage to the cell caused by single-track events, while the β term results from the interaction between two sub-lethal events. The α and β parameters, along with the α/β ratio are found in Table 2. As expected, no effect on the α parameter was found between CTRL and GNP-PEG treated samples (Δ: −0.15, 95% CI: [−0.34, 0.06]; *p* = 0.16). A slight but not significant increase in the α parameter was found in GNP-PEG-RGD treated samples (Δ: −0.18, 95% CI: [−0.37, 0.008]; *p* = 0.06). For both functionalization strategies, no effect on the β parameter was found (GNP-PEG: *p* > 0.05; GNP-PEG-RGD: *p* = 0.97). These results indicate that the primary mechanism of action for GNP-induced radiosensitization could come from increased damage due to single track events, which could be related to an increased generation of secondary electrons. In our previous work, we demonstrated a dose rate effect during 192-Ir source decay, whereby lower source activities resulted in reduced radiosensitization by spherical GNPs.^55^ Given the source activity at the time of irradiation was 7.6 Ci, a higher source activity may have led to a measurable significant increase in the α parameter.

**Table 2:** α and β Parameters from Linear-Quadratic Model Fit to the Clonogenic Survival Data. 95% Confidence Intervals (CI) Provided by Bootstrap Analysis

	CTRL	GNP-PEG	GNP-PEG-RGD
α (*95% CI*)	0.54 (*0.38, 0.70*)	0.69 (*0.56, 0.79*)	0.72 (*0.63, 0.81*)
β (*95% CI*)	0.09 (*0.06, 0.13*)	0.05 (*0.03, 0.08*)	0.09 (*0.07, 0.13*)
α / β (*95% CI*)	6.1 (*2.9, 11.8*)	13.7 (*7.0, 24.0*)	7.8 (*4.9, 12.3*)

Similar results to 2-D cell viability experiments were found measuring spheroid growth (Figure 6D). Without radiation, no effect on the spheroid growth was found after dosing with either GNP-PEG (Rel. Growth: 1.12; *p* = 0.2) or GNP-PEG-RGD (Rel. Growth: 1.14; *p* = 0.41), indicating no toxicity due to the nanoparticle complexes. Post-4 Gy irradiation, no growth suppression was observed in GNP-PEG treated spheroids compared to irradiated CTRL samples (Rel. Growth 1.15; *p* = 0.09). GNP-PEG-RGD treated spheroids did exhibit significant 57% reduction in spheroid growth (*p* < 0.0001), 14 days post-irradiation, compared to CTRL, and a 65% reduction (*p* < 0.0001) compared to GNP-PEG treated samples. This result follows the 2-D monolayer results whereby lower uptake of GNP-PEG presumably led to limited radiosensitization, while RGD-functionalization resulted in high uptake of the nanoparticle and a proportional increase in the measured radiosensitization.

Compared to 2-D monolayer cultures, the relative increase in GNP uptake in 3-D spheroids with RGD functionalization is not on the same order of magnitude, which leads to concern regarding the direct mechanism of action in spheroids of the functionalized GNPs. Spheroids dosed with GNP-PEG-RGD could lead to reduced stromal diffusion of the nanoparticle complexes due to the integrin interaction with proteins and other growth factors present in the spheroid ECM. Without RGD functionalization, a greater number of cells is available for endocytosis via PEGylated GNPs, which could artificially inflate the total uptake compared to if both complexes penetrated the spheroid uniformly. With the current assay and uptake studies, these effects are not able to be separated but will be investigated in future research to quantify the stromal diffusion of functionalized GNPs.

The diameter of the spheroids at the time of irradiation was carefully chosen to limit the formation of a hypoxic and necrotic core that could impact radiosensitization and GNP uptake.^21^,^56^ Shown in Figure S4 are images of PC3 spheroids stained with a hypoxia marker, Image-iT Hypoxia Reagent (Invitrogen), between 3 and 9 days post-seeding showing no hypoxic core formation on the day of irradiation.

### In vivo Radiosensitization

**Figure 7:**
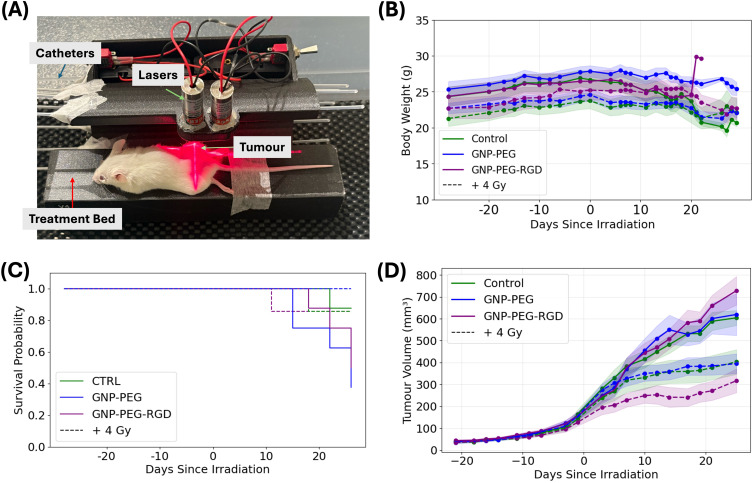
(**A**) Irradiation treatment setup of tumour-bearing mice. Bodyweight change (**B**), overall survival (**C**), and tumour volume growth (**D**) of unirradiated and irradiated mice. Shaded regions in (**B** and **D**) represents ±1 σ/√N.

Treatment delivery of tumour-bearing mice is shown in Figure 7A. At the time of irradiation, the source activity was 8.6 Ci, which resulted in a treatment delivery time of 14 minutes, including dummy source cable checks. The treatment was well-tolerated by the mice, with no immediate signs of stress or observable toxicity noted by the animal care specialists post-irradiation. All mice recovered well from the anaesthesia and were transported back to the animal care facility for monitoring of observable toxicity and tumour growth. Metrics were monitored for up to 40 days post-irradiation, which was the intended endpoint of the experiment. However, data is presented up to 25 days post-irradiations as it was found past this point tumour growth became sporadic between groups with observable metastases in some mice. From the irradiated groups, one mouse from CTRL, one mouse from GNP-PEG treated tumours, and 2 mice from GNP-PEG-RGD treated tumours were removed from the data analysis as their tumours exhibited significant growth rates early on and had to be euthanized shortly after radiation delivery.

Bodyweight change, shown in Figure 7B, pre- and post-irradiation was not statistically significant between groups (all *p* values relative to CTRL > 0.05). Time to endpoint analysis, where the defined event was planned euthanasia upon the tumour volume reaching 800 mm^3^, showed no significant difference (*p* > 0.05) in endpoint-free survival between groups (Figure 7C), indicating that radiation delivery and GNP administration did not accelerate tumour progression to the humane endpoint or induce abnormal toxicity. No statistically significant effect on tumour growth was measured due to the injection of either GNP-PEG (*p* = 0.93) or GNP-PEG-RGD (*p* = 0.93), indicating limited acute toxicity at the delivered dosing concentrations (Figure 7D). Over the course of the experiment, no observable signs of acute toxicity were noted by the animal care specialists, indicating that the retention of the nanoparticle complexes in the mice did not elicit immediate observable toxicity concerns over the course of the experiment.

Irradiated tumours showed significant tumour growth delay compared to unirradiated tumours (Figure 7D). Irradiated CTRL and GNP-PEG treated tumours did not show different growth rates over time, indicating insufficient radiosensitivity from the untargeted nanoparticle complex (*p =* 0.43). GNP-PEG-RGD treated tumours did exhibit statistically significant growth delay compared to both CTRL (Rel. Growth: 0.72; *p* = 0.005) and GNP-PEG (Rel. Growth: 0.71; *p* = 0.04) samples, indicating effective radiosensitization achieved using targeted GNPs. Individual bodyweight and tumour volumes of treatment groups over the study period are shown in Figure S5.

## Discussion

The goal of this research was to systematically evaluate GNP-induced radiosensitization both in vitro and in vivo in HDR-BT. To accomplish this, novel radiation jigs that were previously developed to facilitate in vitro and in vivo HDR-BT irradiations were used to evaluate the efficacy of dual-functionalized GNPs. To the authors’ knowledge, this represents the first investigation of dual-functionalized GNP radiosensitization in vivo from HDR-BT irradiations with a clinically informed i.t. delivery. Prior in vitro work has demonstrated the potential of GNPs in HDR-BT settings, but systematic evaluation in 2-D, 3-D, and in vivo models had not yet been performed. The results presented here have direct implications for the clinical translation of GNPs into HDR-BT workflows by establishing that intracellular localization through RGD-mediated intracellular uptake is a critical determinant of radiosensitization efficacy.

The dosing concentrations employed in this study were chosen to improve the clinical relevance of the GNPs. The desired in vitro dosing concentration of 10 μg [Au]/mL was chosen as this concentration has been shown in previous publications to not inhibit cellular growth in a wide array of phenotypes.^17^,^32^,^41^,^57^ The in vivo dosing concentration of 2 mg [Au]/kg was selected to approximate the in vitro concentration under the assumption that mice weigh approximately 20 g and have a blood volume of approximately 2 mL. Therefore, a dose of 2 mg [Au]/kg bodyweight would yield an approximate peak blood concentration of 10 μg/mL, consistent with the in vitro dosing conditions. Furthermore, the in vivo dose matches previously published research, and is well below the hypothesized LD50 for mice.^32^,^58^ At these dosing concentrations, Monte Carlo simulations predict that little to no dose enhancement should be observed, which contrasts with the presented results.^19^,^59–61^ It has been widely thought that GNPs do not act only on physical dose enhancement, but provide radiosensitization through biological factors as well, such as through mitochondrial depolarization and reactive oxygen species generation.^29–31^ Regardless of their true mechanism of action, we have successfully measured GNP-induced radiosensitization in vivo, with results matching that which was measured in vitro using clinically plausible dosing concentrations that exhibit minimal observable toxicity.

GNP concentration within the tumour was measured to be similar in magnitude between both GNP-PEG and GNP-PEG-RGD treated tumours, similar to other published results.^21^ The question remained whether the complexes were being endocytosed by the tumour cells or if the GNPs were restricted to the extracellular matrix and tumour microenvironment. Our results here indicate that the untargeted GNP-PEG complex could not sufficiently enter the cells in the tumour and were retained in the tumour stroma; however, GNP-PEG-RGD likely did not remain in the tumour stroma and ended up in tumour cells where they can elicit radiosensitization. As previously discussed, the mechanism of action of GNPs is only applicable during their intracellular localization; therefore, purely having the GNPs within tumour stroma is not sufficient to demonstrate their efficacy. It is recommended that in instances where the absolute nanoparticle content in the tumour is measured in biodistribution studies, it should be carefully considered whether the nanoparticle complex is being endocytosed by the tumour cells or if they are being retained in the extracellular matrix. Based on the mechanism of action of certain nanoparticles, either may be beneficial, and we recommend a systematic evaluation of in vitro experiments prior to their introduction into the in vivo environment to determine their uptake and retention kinetics.

Beyond matching the invasive nature of HDR-BT, i.t. delivery of GNPs could also fit within the existing HDR-BT treatment workflows. Grid templates already used for catheter insertion could be repurposed to enable facile needle injection into optimal tumour locations prior to treatment. However, spatial distribution of GNPs within larger human tumours following i.t. injection may be more localized than in the xenografts used here, though complete tumour coverage of GNPs may not be necessary and injection sites could naturally sit in regions of low-dose valleys. Our previous investigations showed excellent tumour retention 48 hours post injection which is a clinically relevant time frame with regards to HDR-BT, indicating that a single pre-treatment injection could maintain adequate GNP concentrations throughout a typical HDR-BT treatment course without requiring repeated administrations.^21^ Future pre-clinical and clinical investigations should address injection site optimization and intratumoural spatial distribution to maximize the therapeutic potential of this approach.

The source activity of 8.6 Ci at the time of irradiation could have impacted the measured radiosensitization. It has previously been shown in published work that GNP-induced radiosensitization exhibits a dose-rate dependency.^62–64^ Therefore, the measured radiosensitization near the start and end of the source’s clinical lifetime may impact the measurable radiosensitization from GNPs. The source activity was chosen to allow for rapid irradiations that did not require excessive anaesthesia for the mice; however, if the irradiations were performed with a lower source activity foregoing anaesthesia safety concern for the animals, perhaps a different radiosensitization would have been measured. However, the current in-vivo irradiation platform may be unable to support longer irradiations from a weak source.

While the results presented here are promising and lay a foundation for future clinical trials, the in vivo safety assessment presented is limited to observable toxicity endpoints like body weight stability monitored by animal care specialists and preliminary blood chemistry at 24 hours post-injection. Blood chemistry showed elevated ALT and AST in the GNP-PEG-RGD treated mouse compared to the PEGylation-only mouse, suggesting acute hepatocellular stress that could be driven by RGD-mediated endocytosis. No other biomarkers, electrolytes, or proteins were found to be significantly different between the two mice. These findings are in contrast to a previous study that investigated long-term toxicity of intravenously injected 20 nm GNPs (1 mg [Au]/kg) and found no elevated liver enzyme change until 60 days post-treatment.^65^ However, their GNPs were not functionalized with active targeting moieties like RGD, which suggests that the RGD-mediated uptake could accelerate the onset of inflammatory stress. Kidney function was also not immediately affected via BUN concentration measurements but may be impacted over long durations.^65^ Yang et al^38^ investigated short-term toxicity of GNP-PEG-RGD intravenously injected (1 mg [Au]/kg) into SCID mice and showed no acute liver toxicity via elevated AST/ALT levels. The twice higher dose and i.t. delivery route possibly leading to concentrated GNP burden during tumour leakage immediately post-injection could be causing this discrepancy, though it is currently unclear given the single animal per group in this study. These findings warrant further investigation with larger cohorts and longer duration studies of RGD-functionalized GNPs.

Similar to the long-term toxicity limitations discussed above, the long-term efficacy of the GNP complexes is also currently unknown and this study design is limited due to its premature termination. This study was not designed to fully address the long-term treatment effects due to inherent limitations of in vivo tumour growth necessitating humane endpoint euthanasia at 800 mm^3^. Future investigations prior to clinical implementation in HDR-BT plans should investigate repeated delivery in fractionated regimens to more accurately reflect clinical treatment plans and determine long-term therapeutic efficacy.

An important limitation of the NRG mouse model employed in this study is the lack of a functional adaptive immune response. It is established that ionizing radiation exerts immunomodulatory effects, which can significantly contribute to therapeutic outcomes in immunocompetent settings, such as the abscopal effect.^66^,^67^ The potential synergy between GNP radiosensitization and radiation-induced immune modulation, therefore, remains unexplored in this study and represents an important avenue for future investigation using immunocompetent mouse models to better replicate the clinical immune environment.

## Conclusion

Gold nanoparticles are a promising radiosensitizing agent for various forms of cancer radiotherapy. Their implementation into high dose rate brachytherapy may be advantageous due to the low-to-medium energy photon emission from the radioactive sources. In this study, we have demonstrated that GNPs functionalized with targeting ligands can induce significant radiosensitization both in vitro and in vivo using clinically relevant dosing concentrations. Both in vitro and in vivo the desired dosing concentrations of the GNPs did not elicit observable toxicity. In vitro, we found that GNPs can increase cellular radiosensitivity by 18% and reduce spheroid growth by 57%. In vivo, functionalized GNPs reduced tumour growth by 28% relative to untreated tumours up to 25 days post-irradiation. Our results are the first of its kind to show GNP-induced radiosensitivity in vivo from a high dose rate brachytherapy source. These findings support the potential of GNPs as effective radiosensitizers for clinical use, highlighting the need for additional research into their safety profile and optimal dosing parameters in humans.

## Data Availability

Data used in the manuscript can be provided upon reasonable request to the corresponding author.

## Abbreviations

EBRT, external beam radiotherapy; GNP, gold nanoparticle; HDR-BT, high dose rate brachytherapy; PEG, polyethylene glycol; STEM, scanning transmission electron microscopy; DLS, dynamic light scattering; UV-VIS, ultraviolet visible spectrometry; PFA, paraformaldehyde; HSI, hyperspectral image; DSB, double strand break; PE, plating efficiency; SF, survival fraction; SER, sensitization enhancement ratio; LQ, linear-quadratic; PDMS, polydimethylsiloxane; A_app_, apparent activity; MC, Monte Carlo.
